# Blastomere removal from cleavage-stage mouse embryos alters placental function, which is associated with placental oxidative stress and inflammation

**DOI:** 10.1038/srep25023

**Published:** 2016-04-25

**Authors:** Qi Yao, Li Chen, Yuanjiao Liang, Liucai Sui, Li Guo, Jingwei Zhou, Kai Fan, Jun Jing, Yunhai Zhang, Bing Yao

**Affiliations:** 1 Center of Reproductive Medicine, Jinling Hospital, Nanjing University School of Medicine, 305 East Zhongshan Road, Nanjing 210002, PR China; 2 Anhui Provincial Laboratory for Local Livestock and Poultry, Genetic Resource Conservation and Breeding, College of Animal Sciences and Technology, Anhui Agricultural University, 130 Changjiang West Road, Hefei 230036, PR China

## Abstract

Blastomere biopsy is an essential technique in preimplantation genetic diagnosis (PGD), a screening test that can detect genetic abnormalities of embryos before their transfer into uterus. Our results showed that the weights of fetuses derived from biopsied embryos were lower than that of non-biopsied counterparts at E12.5, E15.5, and E18.5. The ratio of fetal/placental (F/P) weights in the biopsied group was significantly lower than that in the non-biopsied group at E18.5. At E18.5, the mRNAs for selected glucose transporters, system A amino acid transporters, system L amino acid transporters, and imprinted genes were downregulated in the placentae of biopsied group, and the GLUT1 and CAT3 protein levels were decreased too. More apoptotic cells were detected by TUNEL in the placentae of biopsied group. Placentae from biopsied embryos exhibited lower levels of SOD and GSH. Furthermore, the concentration of MDA increased in the placentae from biopsied group. The levels of IL1B, IL6, and TNFA also significantly increased in the placentae of biopsied group. This study suggested that placental function may be sensitive to blastomere biopsy procedures, and placental oxidative stress and inflammation associated with blastomere biopsy may be critical factors of abnormal placental function and further influence the fetal development.

Since the first successful birth from *in vitro* fertilization (IVF) in 1978, assisted reproductive technologies (ARTs) have been widely used in the treatment of human infertility^1^. According to some statistics, the number of children born through ART has increased to over 5 million worldwide^2^, consisting of an important part of the population. However, some processes involved in ART-mediated conception are very different from natural conception, such as ovarian hyperstimulation, gamete manipulation, preimplantation culture, cryopreservation, and embryo transfer. According to the Developmental Origins of Health and Disease (DOHaD) hypothesis^3^, these techniques perceived by the embryo as stressors may have additional subtle effects that would appear as children grow. Because the eldest IVF individual has just reached her mid-30s, animal models become especially important in determining potential consequences of ART in adulthood. Compared with mice produced by natural conception, mice born through ART are indeed at higher risks of age-related disorders, such as neurodegenerative disorder, cardiovascular disease, metabolic syndrome, and hypertension^4^,^5^,^6^,^7^. Nevertheless, how ART affects fetal programming to elevate the risk of age-related disorders in offspring is still largely unknown.

The placenta is a temporary endocrine gland that transports nutrients and oxygen from the mother to the fetus, playing crucial developmental functions as an interface between the mother and the developing fetus. In response to the changes of resource availability and maternal ecology, placental interface structure and function are regulated by the fetus throughout pregnancy^8^,^9^. It is suggested that placental alterations during gestation are not just a consequence of ART perceived by the embryos, but also play an important role in the impact of ART on fetal programming. Several recent studies have shown that ART procedures and preimplantation embryo culture conditions can alter fetal and placental growth curves as well as the nutrient transport and steroid metabolism in placental tissue^10^,^11^,^12^,^13^,^14^. Therefore, placental phenotype is responsive to the developmental stress of the fetus and may help predict the risk of adult diseases programmed in uterus.

PGD technique removes a single blastomere from an early stage embryo to detect embryonic genetic defects by single cell molecular genetic analyses^15^. This procedure is efficient in assisting reproductive treatment and reducing birth defects. In comparison with other ARTs, the protocol required by PGD not only includes superovulation and *in vitro* embryo culture, but also contains more invasive biopsy procedure of removing 1 or 2 blastomeres from the embryo. Animal studies have demonstrated that mice born through blastomere biopsy are at a higher risk of late-onset neurodevelopmental and metabolic diseases^16^,^17^,^18^,^19^,^20^. Unfortunately, the evaluation of the influence of blastomere biopsy on morphological and functional adaptations in the placenta is scarce.

In this study, we demonstrated that blastomere biopsy adversely affected the subsequent fetal and placental development. Although no structural abnormality was observed in placenta from the biopsied embryos, further testing revealed significantly higher levels of placental cell death (apoptosis) and compromised placental gene expression in the biopsied group. Furthermore, blastomere biopsy manipulation resulted in an increased level of inflammation and oxidative stress in relevant placenta.

## Results

### The Effects of Blastomere Biopsy on Early Embryo Development

**Figure 1:**
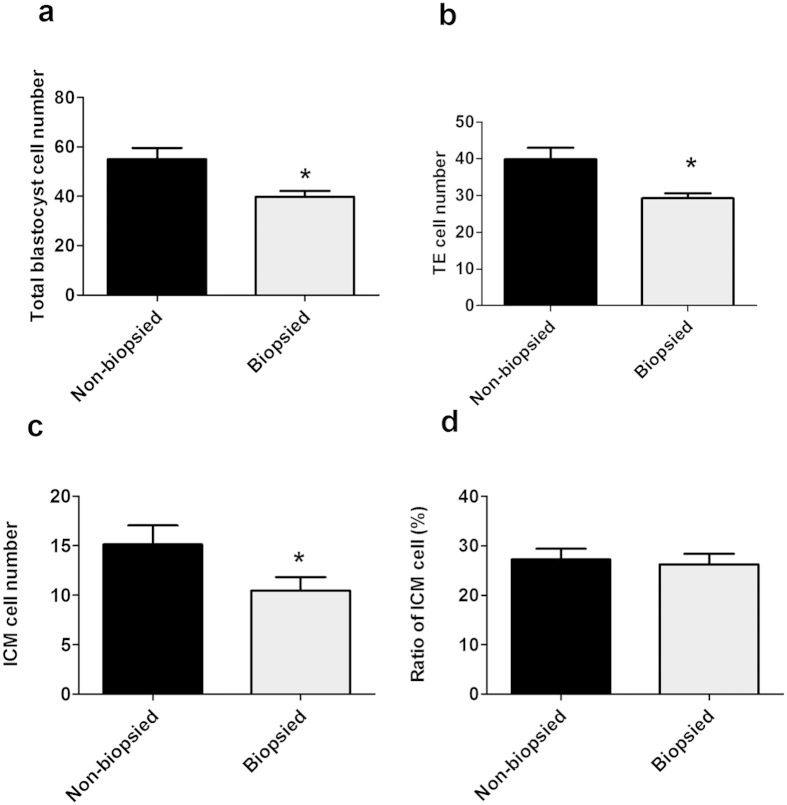
Blastocyst quality by differential staining (n = 15 per group). (**a**) The number of total blastocyst cells. (**b**) The number of TE cells. (**c**) The number of ICM cells. (**d**) The ratio between ICM cell number and the total cell number. Asterisks indicate the statistical significance (*P < 0.001), *VS*. non-biopsied group.

To evaluate the effects of blastomere biopsy on the development of early mouse embryos, 110 embryos at 4-cell stage were involved in blastomere biopsy, of which 105 biopsied embryos were survived, while 103 embryos at 4-cell stage without blastomere biopsy served as control. The blastocyte competence did not differ between the biopsied and non-biopsied groups (*P* > 0.05). However, the total cell number, the cell number of inner cell mass (ICM) and trophoblast (TE) in biopsied embryos decreased significantly (*P* < 0.001, n = 15 per group), though the ratio between ICM cell number and the total cell number was unchanged (*P* > 0.05, n = 15 per group) (Fig. 1).

### The Effects of Blastomere Biopsy on Litter Size and Weights of Fetus and Placenta

The fetal weights in the biopsied group were respectively 10%, 6.4%, and 5.5% lower than those in the non-biopsied group at E12.5, E15.5, and E18.5, (all *P* < 0.05) (Table 1). Of note, there was no significant difference in the average litter size and maternal weight gain (*P* > 0.05) between the biopsied and non-biopsied groups from E12.5 to E18.5 (Table 1). There was no significant difference in the placental weights (*P* > 0.05) between the biopsied and non-biopsied groups from E12.5 to E18.5 (Table 1). However, at E18.5, the F/P weight ratios in the biopsied group were significantly lower than those in the non-biopsied group (*P* < 0.01) (Table 1).

### The Effects of Blastomere Biopsy on Placental Structure

**Figure 2:**
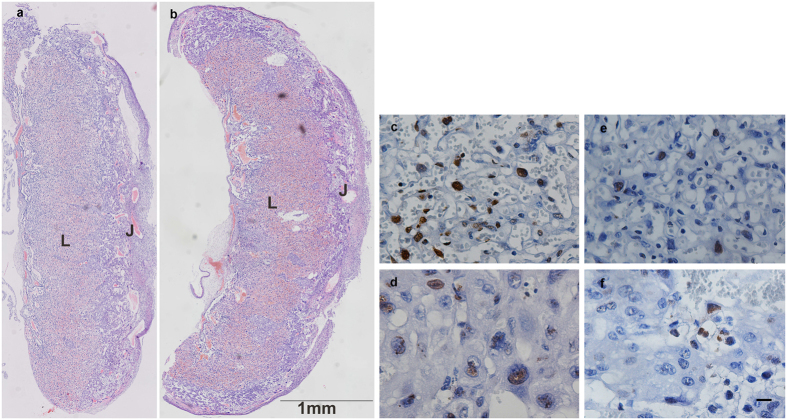
Placental morphology at E18.5. (**a**,**b**) Cross-sections of the entire placenta at E18.5, from non-biopsied group (**a**) and biopsied group (**b**) stained with H&E. L, labyrinth zone; J, junctional zone. (**c**–**f**) Representative microscopic views of placentae apoptosis measured by TUNEL staining in the labyrinth and junctional zone. Labyrinth zone from biopsied placenta (**c**); Junctional zone from biopsied placenta (**d**); Labyrinth zone from non-biopsied placenta (**e**); Junctional zone from non- biopsied placenta (**f**). (Scale bars: 10 μm).

**Figure 3:**
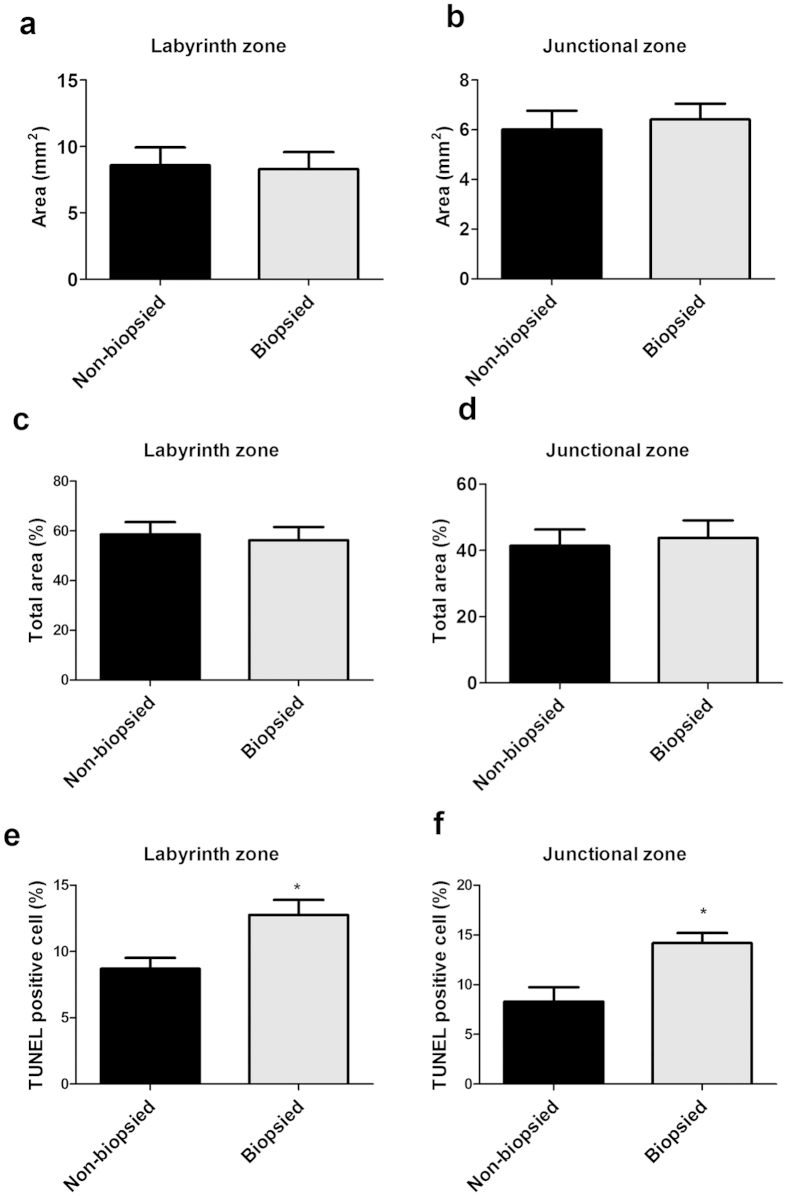
Labyrinth and junctional areas and placental apoptosis at E18.5 (n = 6 per group). (**a**) The area of labyrinth zone. (**b**) The area of junctional zone. (**c**) The ratio between labyrinth zone area and whole placenta area. (**d**) The ratio between junctional zone area and whole placenta area. (**e**) The percentage of TUNEL positive cells in labyrinth zone. (**f**) The percentage of TUNEL positive cells in junctional zone. Asterisks indicate the statistical significance (*P < 0.001), VS. non-biopsied group.

No obvious histological change in the placentae, such as changes in fibrin deposits, syncytial knots, or overt endothelial changes, was observed under a light microscope. The gross morphology of placenta did not differ between both groups at E18.5 (Fig. 2a,b). The areas of the labyrinth and junctional zone were not different (*P* > 0.05, n = 6 per group) (Fig. 3). TUNEL staining with DAB detection showed that higher levels of apoptosis were observed visually in both the labyrinth and junctional zone in biopsied group, compared with non-biopsied group (*P* < 0.001, n = 6 per group) (Figs 2c–f and 3).

### The Effects of Blastomere Biopsy on the Expression of Selected Transporters and Imprinted Genes in the Placentae at E18.5

**Figure 4:**
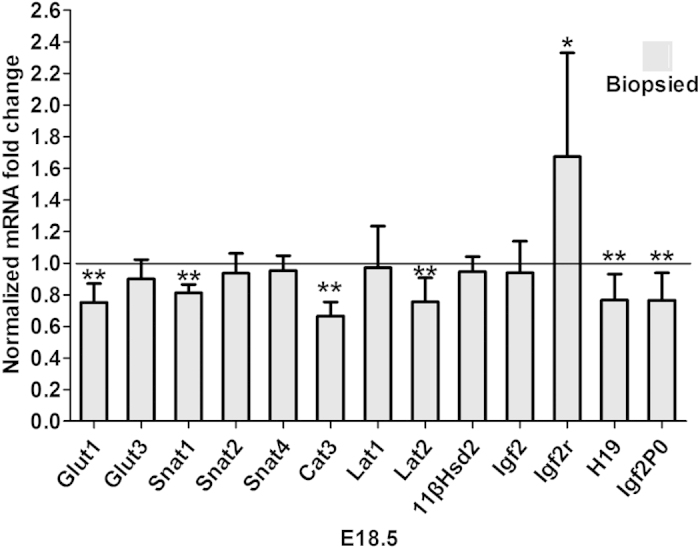
The expression of selected transporter genes and imprinted genes in placentae at E18.5. Real-time PCR for the relative amounts of Glut1, Glut3, Snat1, Snat2, Snat4, Cat3, Lat1, Lat2,Igf2, Igf2r, H19, Igf2P0, and11βHsd2 mRNA in total placental tissue at E18.5 in the non-biopsied and biopsied groups (n = 6 per group). Asterisks indicate the statistical significance (*P < 0.05, **P < 0.01)*VS*. non-biopsied group.

To evaluate the effects of blastomere biopsy on the expression of placental nutrient transporters, we used real-time quantitative PCR to investigate the changes in the expression of placental nutrient transporters at E18.5 (non-biopsied group, 18 placentae of 6 litters; biopsied group, 18 placentae of 6 litters). The average cycle threshold value of the three placentae from one litter was used for statistical analysis. Our results revealed that the glucose transporter Glut1, the neutral amino acid transporter Snat1 (Slc38a1), and the cationic amino acid transporters Cat3 (Slc7a3) and Lat2 (Slc7a8) were significantly downregulated (*P* < 0.01, n = 6 per group) (Fig. 4).

**Figure 5:**
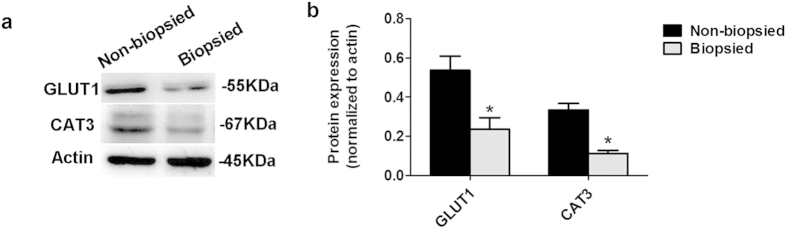
The expression of GLUT1 and CAT3 protein in placentae at E18.5. (**a**) Representative Western blottings of GLUT1, CAT3, and Actin in total placental homogenates, obtained from the non-biopsied and biopsied groups (3 litters per group). (**b**) Densitometric measurements of Western blotting autoradiograms (n = 3 per group). Asterisks indicate the statistical significance (*P < 0.001)*VS*. non-biopsied group.

To better understand the significance of the gene expression findings, we quantified GLUT1 and CAT3 protein expressions using commercially available antibodies, because these 2 transporters had the most distinctive changes among placental glucose and amino acid transporter genes observed. The decrease in Glut1 mRNA and Cat3 (Slc7a3) mRNA expressions at E18.5 was associated with a decreased protein expression in whole placentae of the biopsied group (*P* < 0.001, 3 litters per group) (Figs 5 and S1).

Next, we examined whether or not there were differences in the expression of specific imprinted genes in placentae, and the results showed that Igf2p0 (IGF2 placental-specific isoform) and H19 were downregulated (*P* < 0.01, n = 6 per group), whereas the Igf2r was significantly increased in the placentae of biopsied group (*P* < 0.05, n = 6 per group) (Fig. 4).

Finally, as blastomere biopsy has been found to impair placental steroid metabolism^21^, we evaluated 11βHsd2 mRNA level in placentae and found that the expression of 11βHsd2 mRNA in biopsied group was similar to that in non-biopsied group at E18.5 (*P* > 0.05, n = 6 per group) (Fig. 4).

### The Effects of Blastomere Biopsy on Oxidative Stress and Antioxidant Ability in Maternal Livers, Fetal Livers, and Placentae at E18.5

The levels of malondialdehyde (MDA), a product of lipid peroxidation, were significantly increased in placentae from biopsied group compared with non-biopsied group. Meanwhile, the levels of antioxidant substances, superoxide dismutase (SOD) and glutathione (GSH), were significantly decreased in placentae from biopsied group compared with non-biopsied group (*P* < 0.001) (Table 2).

No significant difference in antioxidant ability was observed in maternal livers (*P* > 0.05) between biopsied group and non-biopsied group (Table 2).

In fetal livers from biopsied group, antioxidant enzyme capacity for catalase (CAT) was significantly reduced compared with non-biopsied counterparts, while antioxidant enzyme capacity for SOD was significantly increased (*P* < 0.001) (Table 2).

### The Effects of Blastomere Biopsy on the Levels of Proinflammatory Cytokines in Maternal Livers, Fetal Livers, and Placentae at E18.5

The levels of 3 major proinflammatory cytokines (IL6, TNFA, and IL1B) were measured in maternal livers, fetal livers, and placentae. Higher levels of IL6, TNFA, and IL1B were observed in placentae from biopsied group compared with non-biopsied group (*P* < 0.05) (Table 3). However, there was no difference in the levels of these 3 proinflammatory cytokines in maternal and fetal livers (*P* > 0.05) (Table 3).

## Discussion

This study demonstrated that blastomere biopsy affected the fetal and placental development of Kunming white mice. Compared with non-biopsied group, the weights of biopsied embryos were lower at E12.5, E15.5, and E18.5, while a compensatory growth in size was achieved from E12.5 to E18.5. In addition, the placental weights of biopsied group were similar to that of non-biopsied group at the same gestational age points from E12.5 to E18.5. However, the ratio of placental weight between the biopsied and non-biopsied groups increased progressively during the gestation period. We speculated that the limited growth in biopsied placentae cannot meet the demand of biopsied fetal catch-up growth. It is worth noting that the F/P weight ratio in the biopsied group was significantly lower than that in the non-biopsied group at E18.5. The F/P ratio is considered as a marker of intrauterine stress^21^. It has been shown that intrauterine stress continues to affect the postnatal health. A decreased F/P ratio has been described in human pregnancies to be associated with cardiovascular disease in later life^21^,^22^,^23^,^24^. Therefore, these altered growth curves for biopsied fetuses and their placentae suggested that the embryos underwent preimplantation blastomere biopsy were suboptimal.

Placenta is a key organ for fetal growth. This study showed that there was no histologic abnormality in placentae from pregnancies with embryos received blastomere biopsy. Sugawara reported that placental steroid metabolism was dysregulated in mouse pregnancies by embryos underwent blastomere biopsy^25^. Thus, we hypothesized that placental malfunction might be important in growth retardation of biopsied embryos/fetuses. Appropriate fetal growth and development are largely determined by adequate nutrient supply which is dependent upon placental nutrient transport. In mammalian pregnancies complicated by either intrauterine growth restriction (IUGR) or fetal overgrowth, many key placental nutrient transporters are specifically regulated^8^,^9^,^26^. There are two principal glucose transporters, Glut1 and Glut3, in placenta. In this study, we found that the levels of Glut1 were decreased in placentae of biopsied group at E18.5. Studies have demonstrated that Glut1 expression can be epigenetically regulated^27^,^28^, and blastomere biopsy alters epigenetic markers^17^,^19^, suggesting that the changes in Glut1 expression observed in this study might be caused by epigenetic mechanisms.

The placental system A amino acid transporter is composed of three functionally independent protein/gene isoforms of Na^+^-coupled neutral amino acid transporter Snat1/Slc38a1, Snat2/Slc38a2, and Snat4/Slc38a4. These three system A amino acid transporters actively transport small, zwitterionic, and neutral unbranched amino acids such as serine, alanine, and glutamine^29^,^30^. In this study, we found that Snat1 transcripts were downregulated in the placentae of biopsied group at E18.5. Evidence from studies suggests that reduced system A activity may be a cause of fetal growth retardation, or related to altered fetal growth^31^.

The placental system L amino acid transporter is Na^+^-independent but energy-dependent. It mainly transports neutral branched chain amino acids like leucine. The placental system L amino acid transporter has three functionally independent protein/gene isoforms: Cat3/Slc7a3, Lat1/Slc7a5, and Lat2/Slc7a8^32^. In this study, we found that Cat3 and Lat1 transcripts were downregulated in the placentae of biopsied group compared with the non-biopsied control at E18.5. In human pregnancies associated with IUGR, reduced activity of system L amino acid transporter has been reported in the postparturient placenta^33^.

Imprinted genes in mammals are expressed in a parent-of-origin specific way. They are also known to be critically involved in feto-placental development and placental nutrient-transporting capacity^34^,^35^. Expression of a selection of key imprinted genes in the placenta was also examined in the current study, and the results showed that Igf2P0 transcripts in the placentae of biopsied group were downregulated compared with the non-biopsied group at E18.5. The Igf2P0 regulates the placental expression and activity of selected glucose and amino acid transporters in a time-dependent manner^36^,^37^. More specifically, the absence of the P0 transcript in a gene deletion experiment resulted in growth deficiency of the placenta and reduction in permeability for nutrients, leading to fetal growth retardation^38^. In addition, Igf2r transcripts in the placentae of biopsied group were upregulated compared with the control. Igf2r mediates the clearance of Igf2. Studies showed that the decrease of the level of Igf2r in BeWo cells (a human choriocarcinoma cell line) enhanced Igf2-mediated rescue from apoptosis^39^,^40^. Therefore, Igf2 plays a critical role in placental development and function. One limitation of our study is that Igf2 gene expression was observed without Igf2 peptide analysis in placentae. In the present study, the placental mRNA expression of Igf2 was similar in both groups, while the relative mRNA expression of H19 in the placentae of biopsied group decreased significantly. Because H19 and Igf2 share common regulatory elements, in theory, the reduction in total levels of H19 mRNA would be accompanied by a concomitant increase in levels of Igf2 mRNA, which, however, was not observed in this study. One possible reason for the difference in this study is that the total levels of H19 and Igf2 transcriptions were analyzed in the genome, but neither the paternal nor maternal monoallelic expressions were investigated. Rivera et al. found that the total levels of H19 mRNA were not statistically different in manipulated placentae when compared with control placentae^41^. However, when they divided all of the manipulated placentae by maternal or paternal allelic expression, a significant difference was found.

Additionally, more degraded DNA (apoptosis) was detected by TUNEL in the placentae of biopsied group. Increased apoptosis rates have been reported in placentae from cases of IUGR and mouse pregnancies by IVF and intracytoplasmic sperm injection (ICSI)^42^,^43^,^14^. Increased apoptosis could be the beginning of placental functional changes that fail to meet fetal demands required for normal growth, as previously described in mouse model^44^. We speculated that functional placental changes may be partly mediated by apoptotic signaling in mouse pregnancies from biopsied group.

Excessive placental oxidative stress and inflammation were also detected in the biopsied group. Previous research has shown that oxidative stress tends to cause localized apoptosis in the labyrinth zone of the murine placenta^45^. Additionally, increased oxidative stress results in reduced glucose and neutral amino acids uptake and lowered Glut1 expression in placentae^46^,^47^,^48^. Research also showed that both TNFA and IL1B downregulate placental glucose and neutral amino acids uptake and Glut1 expression^47^,^49^. Therefore, oxidative stress and inflammation in placentae may be critical factors for impaired placental function of biopsied group.

In summary, our study provides the first evidence that blastomere biopsy impairs placental function, alters both fetal and placental development, dysregulates placental antioxidant defense, and increases placental inflammation in mice. Our results support that excessive placental oxidative stress and inflammation associated with blastomere biopsy may play important role in placental dysfunction, which may alter fetal development. A deeper understanding of placental dysfunction in PGD will improve PGD outcomes.

## Methods

### Chemicals and Reagents

Pregnant mare serum gonadotropin (PMSG) and human chorionic gonadotropin (hCG) were from Hua Fu Biotechnology Company (Tianjin, China). Quinn’s advantaged^TM^ Medium with HEPES, Quinn’s advantaged^TM^ protein plus fertilization Medium, Quinn’s advantaged^TM^ protein plus cleavage medium, Quinn’s advantaged^TM^ protein plus blastocyst medium, Quinn’s advantaged^TM^ with Ca^2+^/Mg^2+^ free Medium HEPES were from Sage BioPharma. Paraffin oil and hyaluronidase was from Sigma (St. Louis, MO, USA).

### Animals

Mice of Kunming white strain were from the Lab Animal Center in Nanjing Jinling Hospital of China. All experimental procedures involved in the mouse studies were approved by the Committee on the Ethics of Animals and Medicine of the Nanjing Jinling Hospital, which is in accordance with the principles and procedure of the NIH guide for the care and use of laboratory animals. Animal Care Virgin 6- to 8- wk-old Kunming white female mice, adult Kunming white males, and Kunming white vasectomized males (n = 12) were used. Mice were fed *ad libitum* with a standard diet and maintained in a temperature and light-controlled room (22 °C, 14 h light/10 h dark).

### Ovarian Stimulation and Embryo Culture

Female mice were superovulated by i.p. injection of 10 IU (0.1 mL) PMSG, followed by i.p. injection of 10 IU (0.1 mL) hCG 48 h later. After hCG injection, female mice were mated with ten-week-old male mice. Fertilized embryos were collected 20–22 h after hCG injection from the plugged females and cultured until the 4-cell stage in Quinn’s advantaged^TM^ protein plus cleavage medium. Groups of 4-cell embryo were transferred into a droplet of Quinn’s advantaged^TM^ with Ca^2+^/Mg^2+^ free Medium HEPES.

### Cleavage-stage Biopsy and Embryo Transfer

One blastomere in a 4-cell embryo was removed randomly with an enucleation pipette as described previously for human blastomere biopsy^50^. After manipulation, the embryos were cultured until the early blastocyst stage in Quinn’s advantaged^TM^ protein plus blastocyst medium.

Pseudo-pregnant Kunming females were used as embryo recipients after mating with vasectomized Kunming males. Eight early blastocysts from biopsied “3-cell” embryos and control “4-cell” embryos were transferred into left-side uterine horn of 2.5 pesudopregnant Kunming females. The right-side uterine horn served as a control that the vasectomized males were indeed consistently sterile.

### Differential Embryo Staining

TE and ICM cell numbers of non-biopsied and biopsied blastocysts were stained with propidium iodide and Hoechst 33342 as described^51^. Blastocysts were first incubated in Dulbecco’s phosphate buffered saline (D-PBS) with 1% Triton ×100 and 100 μg/ml of propidium iodide for 10 s. The blastocysts were then fixed overnight in 100% ethanol containing 50 μg/ml of Hoechst 33342. Finally, the stained blastocysts were mounted on glass slides in a drop of glycerol, and cell counting was performed from images obtained under an inverted microscope fitted with an ultraviolet lamp and excitation filters (410 and 560 nm for blue and red fluorescence, respectively).

### Placental and Fetal Dissection

Pregnant mice were euthanized by ether inhalation and subsequent cervical dislocation, whereas fetuses were euthanized by decapitation. Fetuses and placentae were harvested at 3 different time points, E12.5, E15.5, and E18.5. After dissection, the placental and fetal wet weights were recorded, and each placenta was immediately fixed overnight in 10% neutral buffered formalin for histological analyses, or placed in RNALater (QIAGEN) for real-time PCR, or frozen in liquid nitrogen for Western blot analysis and biochemical analysis.

### Histological Examination of the Placentae

At E18.5, one placenta, with a wet weight closest to the mean weight of the litter, was selected from each litter (n = 6 per group), bisected through the attachment of the umbilical cord, and embedded in paraffin wax. Cross sections of 4 μm were obtained and stained with hematoxylin and eosin (H&E).

The border between the junctional and labyrinthine zones was identified visually in H&E-stained sections. The border between maternal and fetal components of the placenta was identified by the presence of trophoblast giant cells; the total cross-sectional areas of junctional and labyrinthine zones were estimated using cellSens software (version 1.8, OLYMPUS Inc).

### Histological Examination of Placental Apoptosis

Terminal deoxynucleotidyl transferase dUTP nick end labeling (TUNEL) was performed with an *in situ* detection kit following the manufacturer’s instructions (ROCH). Systematic random sampling was used to select field of view for counting at 400× magnification. The percentage of immunostained cells was estimated by counting approximately 1,300 cells per placenta.

### RNA Extraction, cDNA Preparation, and Real-time PCR Analysis

RNA was extracted independently from each placenta (18 placentae per group). In particular, the 3 placentae with the weight closest to the mean in each litter were used (6 litters per group). The average cycle threshold value of the three placentae from one litter was used for statistical analysis. The tissues were manually homogenized respectively. Total RNA was extracted with RNeasy Protect Mini kit (QIAGEN Inc). Reverse transcription was accomplished by using a commercially available first strand cDNA synthesis kit (FERMENTAS Inc) with 4 μg of total RNA.

Real-time PCR was performed on an Applied Biosystems StepOnePlus^TM^ Real-Time PCR System (Applied Biosystems, Foster City, CA, USA), with oligonucleotide primers synthesized by the INVITROGEN and the SYBR Green PCR Supermix from TaKaRa Inc). The primer sequences for the genes analyzed are listed in Table 4. Genes evaluated were the glucose transporter isoforms *Glut1* and *Glut3,* the system A amino acid transporter isoforms sodium-coupled neutral amino acid transporters *Snat1* (also known as *Slc38a1*), *Snat2* (*Slc38a2*), and *Snat4* (*Slc38a4*), the system L amino acid transporter isoforms *Cat3* (*Slc7a3*), *Lat1*(*Slc7a5*), and *Lat2* (*Slc7a8),* the *11β-Hsd2,* the paternally imprinted genes *Igf2* and *Igf2p0* (IGF2 placental-specific isoform), the maternally imprinted genes *H19* and *Igf2r,* and the housekeeping gene glyceraldehyde 3-phosphate dehydrogenase (*Gapdh*). Duplicates were used for each reaction, and a no-template control was included in all runs using water instead of cDNA. Data were analyzed by the comparative threshold cycle method. The thermal cycle conditions used were as follows: 35 cycles of 95 °C for 15 sec, 60 °C for 30 sec, using 40 ng of cDNA in final reaction volume of 20 μL.

### Western Blotting

The expression of GLUT1 and CAT3 protein was analyzed in placental homogenates. Three placentae from 3 different dams in each group were harvested and processed. The placentae were homogenized and suspended in a lysis buffer (BioDev-Technology, Beijing, China). Twenty μg of protein were resolved by SDS-PAGE (12% gel) and then transferred to polyvinylidene difluoride membrane. After blocking in blocking solution [Tris-buffered saline with 0.1%Tween 20 (TBST) and 5% BSA] for 1.5 h, the membranes were incubated at 4 °C overnight with specific antibodies anti-GLUT1 (1:1000 dilution; ab32551, Abcam) and anti-CAT3 (1:1000 dilution; ab113985, Abcam) respectively in 5% BSA. The membrane was then washed 3 times with TBST, and bands were visualized with the appropriate horseradish peroxidase-conjugated secondary antibodies and the enhanced chemiluminescence system, in accordance with the manufacturer’s instructions.

### ELISA Detection of Inflammatory Cytokines

The concentrations of inflammatory cytokines IL1B, IL6, and TNFA in placentae and maternal and fetal livers were determined using commercial mouse immunoassay ELISA kits (Neobioscience), according to the manufacturer’s instructions. The concentrations of cytokines detected in the samples were determined from standard curves.

### Biochemical Assays for Antioxidants and Oxidants

Superoxide dismutase (SOD), Catalase (CAT), Glutathione peroxidase (GPx), Gluthathione reductase (GR), Glutathione-S-transferase (GST), Glutathione (GSH), and malondialdehyde (MDA) were tested in placentae and maternal and fetal livers from each group, using commercial kits following the manufacturer’s instructions (Nanjing Jiancheng Bioengineering Inc.; the unit was demonstrated as μmolPi/mgprot/hour).

### Statistical Analysis

Values for all data were expressed as mean ± SD. Data were analyzed using unpaired two-tailed Student’s *t*-test for significant difference between the 2 groups with SPSS 17.0 software. P < 0.05 was considered statistically significant.

## Additional Information

**How to cite this article**: Yao, Q. *et al*. Blastomere removal from cleavage-stage mouse embryos alters placental function, which is associated with placental oxidative stress and inflammation. *Sci. Rep.* **6**, 25023; doi: 10.1038/srep25023 (2016).

## Supplementary Material

Supplementary Information

## Figures and Tables

**Table 1:** Litter size and fetal and placental weight.

	Age	Litters	Maternal Weight gain (g)	Litter size	Placental weight (mg)	Fetal weight (mg)	F:P ratio
Non-biopsied	12.5	3	12.4 ± 1.6	7.3 ± 0.6	69.90 ± 12.39	71.48 ± 10.02	1.04 ± 0.22
Biopsied	12.5	3	10.4 ± 1.5	6.0 ± 1.0	70.47 ± 14.04	**64.36** ± 11.52*	0.94 ± 0.25
Non-biopsied	15.5	3	13.1 ± 1.1	6.3 ± 0.6	142.95 ± 12.59	417.89 ± 23.92	2.94 ± 0.26
Biopsied	15.5	3	11.8 ± 1.2	5.7 ± 0.6	144.59 ± 15.34	**391.37** ± 40.48*	2.73 ± 0.38
Non-biopsied	18.5	6	21.6 ± 2.9	6.5 ± 1.4	179.31 ± 24.72	1653 ± 137	9.35 ± 1.16
Biopsied	18.5	6	18.4 ± 3.5	5.3 ± 1.4	188.70 ± 23.70	**1560** ± 170*	**8.42** ± 1.53**

Statistical significance: ^*^P < 0.05, ^**^P < 0.01, *VS*. non-biopsied group, and significant changes are bolded.

**Table 2:** Oxidative stress and antioxidant ability in the placentae and maternal and fetal livers at E18.5.

Tissue	Treatment	SOD U/mgprot	CAT U/mgprot	GSH-PX U/mgprot	GR U/mgprot	GST U/mgprot	GSH μmol/gprot	MDA nmol/gprot
Maternal liver	Non-biopsied(n = 6)	136.08 ± 9.80	9.38 ± 0.81	414.31 ± 61.29	5.50 ± 0.98	19.81 ± 2.84	2.46 ± 0.33	1.78 ± 0.16
	Biopsied(n = 6)	135.91 ± 16.32	9.63 ± 0.37	363.47 ± 24.13	6.17 ± 0.78	19.10 ± 5.14	2.18 ± 0.26	1.73 ± 0.36
Fatal Liver	Non-biopsied(n = 38)	26.64 ± 8.70	4.54 ± 0.57	52.96 ± 10.50	4.81 ± 1.23	35.95 ± 8.23	4.70 ± 1.02	1.52 ± 0.34
	Biopsied(n = 30)	**36.10** ± 6.97*	**3.96** ± 0.55*	49.19 ± 11.20	4.31 ± 1.06	32.69 ± 6.05	5.14 ± 1.17	1.32 ± 0.56
Placenta	Non-biopsied(n = 38)	26.31 ± 4.32	1.04 ± 0.22	45.68 ± 11.51	4.62 ± 1.37	12.83 ± 3.39	2.42 ± 0.63	0.71 ± 0.33
	Biopsied(n = 30)	**14.65** ± 4.54*	1.00 ± 0.24	41.38 ± 10.28	4.06 ± 1.54	12.50 ± 2.70	**1.25** ± 0.54*	**1.03** ± 0.14*

Statistical significance: ^*^P < 0.001, *VS*. non-biopsied tissue, and significant changes are bolded.

**Table 3:** The levels of proinflammatory cytokines in the placentae and maternal and fetal livers at E18.5.

Tissue	Treatment	IL1B	IL6	TNFA
		pg/ml/mg prot	pg/ml/mg prot	pg/ml/mg prot
Maternal liver	Non-biopsied(n = 6)	94.58 ± 4.68	106.90 ± 4.90	127.19 ± 15.36
	Biopsied(n = 6)	94.15 ± 8.36	105.85 ± 9.63	128.80 ± 15.31
Fatal Liver	Non-biopsied(n = 38)	94.37 ± 15.46	120.75 ± 27.37	120.92 ± 23.53
	Biopsied(n = 30)	99.86 ± 13.00	110.30 ± 13.64	103.16 ± 13.32
Placenta	Non-biopsied(n = 32)	14.92 ± 5.57	13.63 ± 5.62	3.92 ± 1.55
	Biopsied(n = 24)	**20.98** ± 10.19*	**18.61** ± 8.04*	**6.38** ± 3.97**

Statistical significance: ^*^P < 0.05, ^**^P < 0.01, *VS*. non-biopsied tissue, and significant changes are bolded.

**Table 4:** Primers used for real-time PCR.

	Sequence
*Glut1*	Forward, CCAGCTGGGAATCGTCGTT Reverse, CAAGTCTGCATTGCCCATGAT
*Glut3*	Forward, CTCTTCAGGTCACCCAACTACGT Reverse, CCGCGTCCTTGAAGATTCC
*Snat1*	Forward, AGAAGTAGAAAACGGCCAGATAAAT Reverse, ATACTTACATACTCGTCGCATTTCC
*Snat2*	Forward, GACTTCCCAAACCTGTG Reverse, CAGCGATGTGAATTGAGGTG
*Snat4*	Forward, TCACACTGCTGTTTCCAAGG Reverse, CAGCCGGAAGAATGAAAATC
*Lat1*	Forward, GCTCTGGCATCTTCGTGAC Reverse, CCGTAGACCTCCAGCATGTA
*Lat2*	Forward, AGTTTCCAACCTCCAGGACA Reverse, CGGTGGTAGGGTTGTTTGAG
*Cat3*	Forward, TTCTGGCCGAGTTGTCTATGTTTG Reverse, AGTGCGGTTCTGTGGCTGTCTC
*11βHsd2*	Forward, CTGCAGATGGATCTGACCAA Reverse, GTCAGCTCAAGTGCACCAAA
*IGF2*	Forward, AAGAGTTCAGAGAGGCCAAACG Reverse, CACTGATGGTTGCTGGACATCT
*Igf2r*	Forward, TTTTGGGCGCCTTGCAT Reverse, AGGGCAAGGATCACCATTCAC
*H19*	Forward, AATGGTGCTACCCAGCTCAT Reverse, GCAGAGTTGGCCATGAAGAT
*Igf2P0*	Forward, CCGAGGCCTGTACCACCTA Reverse, CCTCGGCTCAGACCTCAGTA
*Gapdh*	Forward, ACAACTTTGGCATTGTGGAA Reverse, GATGCAGGGATGATGTTCTG
