# Supplementary material for: Blastomere removal from cleavage-stage mouse embryos alters placental function, which is associated with placental oxidative stress and inflammation
Source: Sci Rep. 2016 Apr 25;6:25023. doi: 10.1038/srep25023 (PMC4842963; doi:10.1038/srep25023)
Supplement: Supplementary Information [file srep25023-s1.doc]

**Blastomere removal from cleavage-stage mouse embryos alters placental function, which is associated with placental oxidative stress and inflammation**

Qi Yao^1^, Li Chen^1^, Yuanjiao Liang^1^, Liucai Sui^1^, Li Guo^1^, Jingwei Zhou^1^, Kai Fan^1^, Jun Jing^1^, Yunhai Zhang^2^, ＆Bing Yao^1^

Q.Y., L.C., Y-J.L., and L-C.S contributed equally to this work.

1: Center of Reproductive Medicine, Jinling Hospital, Nanjing University School of Medicine, 305 East Zhongshan Road, Nanjing 210002, PR China.

2: Anhui Provincial Laboratory for Local Livestock and Poultry, Genetic Resource Conservation and Breeding, College of Animal Sciences and Technology, Anhui Agricultural University, 130 Changjiang West Road, Hefei 230036, PR China.

Corresponding author: Professor Yunhai Zhang, Anhui Provincial Laboratory for Local Livestock and Poultry, Genetical Resource Conservation and Breeding, College of Animal Sciences and Technology, Anhui Agricultural University, 130 Changjiang West Road, Hefei 230036, PR China (FAX: +86-551-25786357; Email: yunhaizhang@ahau.edu.cn)

Corresponding author: Professor Bing Yao, Center of Reproductive Medicine, Jinling Hospital, Nanjing University School of Medicine, 305 East Zhongshan Road, Nanjing 210002, PR China. (FAX: +86-25-80860174; E-mail: 2424572228@qq.com)

**
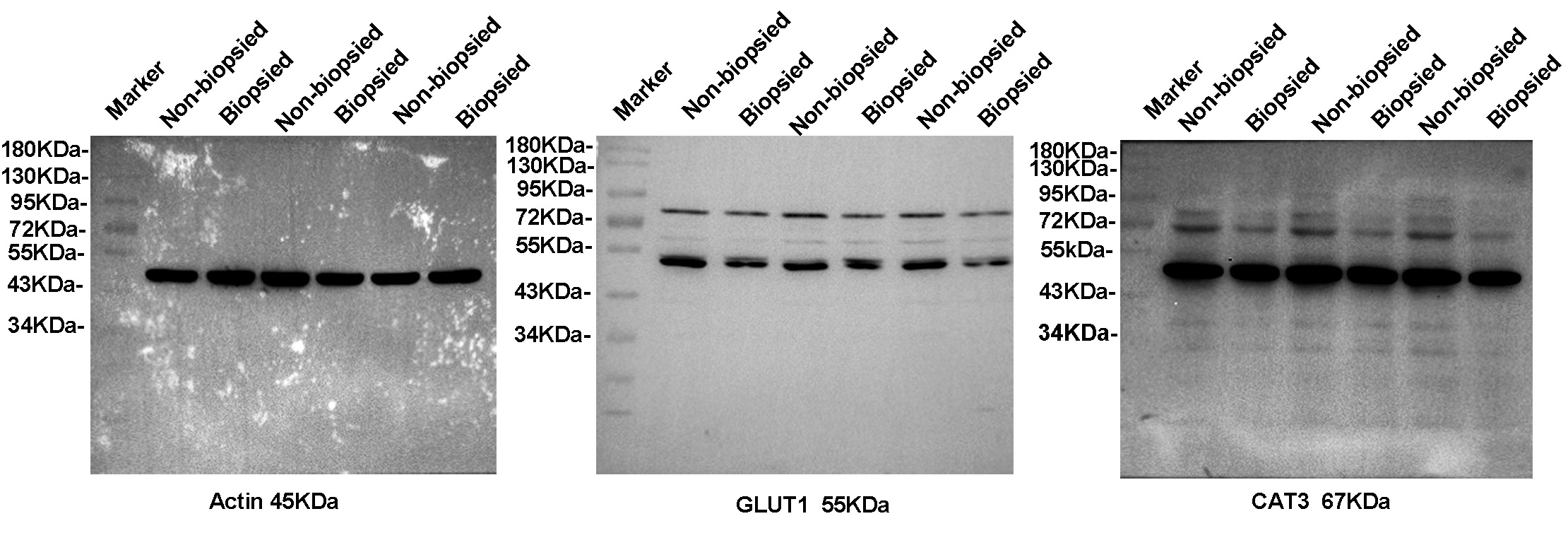
**

**Fig. S1**

Western blottings of GLUT1, CAT3, and Actin in total placental homogenates, obtained from the non-biopsied and biopsied groups (3 litters per group).
